# Correction: “*We know about schistosomiasis but we know nothing about FGS*”: A qualitative assessment of knowledge gaps about female genital schistosomiasis among communities living in *Schistosoma haematobium* endemic districts of Zanzibar and Northwestern Tanzania

**DOI:** 10.1371/journal.pntd.0011099

**Published:** 2023-01-26

**Authors:** Humphrey D. Mazigo, Anna Samson, Valencia J. Lambert, Agnes L. Kosia, Deogratias D. Ngoma, Rachel Murphy, Dunstan J. Matungwa

There is information missing from the Acknowledgments section. It should read as follows:

The authors would like to thank the study participants for their time and responses without which this paper would not have been possible to write. We also thank the regional, district, shehia and village authorities where the study was conducted for granting the permission and providing every support to successfully conduct this study. We are grateful to Prof. Jennifer A. Downs of the Center for Global Health, Weill Cornell Medicine, New York, United States of America for her support in conceptualizing the project whose outputs include this paper. We also thank her for the reviews, comments and suggestions on the early drafts of this article. The authors would like to acknowledge that the article included qualitative data designed and collected in Tanzania as part of the study “Bringing Down Hurdles for Female Genital Schistosomiasis Access to Care: a Multi-Country Socio-Structural Integrated Approach to Developing a Community-Based Teaching Platform” carried out in Zambia, Tanzania and Malawi, funded by The Task Force for Global Health NTD-SD, and led by Virginia Bond (Lead PI, LSHTM and Zambart), Humphrey Mazigo (Catholic Institute for Health and Allied Sciences/Bugando) and Khumbo Kalua (Blantyre Institute for Community Outreach (BICO). The views expressed in this publication are those of the authors and not necessarily those of the funding agencies. Its contents are solely the responsibility of the authors and do not necessarily represent the official views of the supporting offices.
