# Supplementary material for: “We know about schistosomiasis but we know nothing about FGS”: A qualitative assessment of knowledge gaps about female genital schistosomiasis among communities living in Schistosoma haematobium endemic districts of Zanzibar and Northwestern Tanzania
Source: PLoS Negl Trop Dis. 2021 Sep 30;15(9):e0009789. doi: 10.1371/journal.pntd.0009789 (PMC8509863; doi:10.1371/journal.pntd.0009789)
Supplement: Attachment — Submitted filename: Mazigo-FGS KAP Community members_Manuscript_draft_DM (170621)_JD-Rev.doc [file pntd.0009789.s001.doc]

# “*We know about schistosomiasis but we know nothing about Female Genital Schistosomiasis*”: Knowledge gaps about female genital schistosomiasis among communities living in *Schistosoma haematobium* endemic districts of Zanzibar and Northwestern Tanzania

Humphrey D. Mazigo^1*^, Anna B. Samson^2^, Valencia J. Lambert^3^, Agnes Kosia^4^, Deogratias D. Ngoma^5^, Rachel Murphy^6^_,_ Dunstan J. Matungwa^7, 8^

^1^Department of Parasitology and Entomology, Weill Bugando School of Medicine, Catholic University of Health and Allied Sciences, Mwanza, Tanzania

^2^Department of Behavioral Sciences, School of Public Health, Catholic University of Health and Allied Sciences, Mwanza, Tanzania

^3^Center for Global Health, Weill Cornell Medicine, New York, United States of America.

^4^School of Nursing, Catholic University of Health and Allied Sciences, Mwanza, Tanzania

^5^Accelerating the Sustainable Control and Elimination of Neglected Tropical Diseases Project

^6^ Crown Agents, London, United Kingdom

^7^Department of Sexual and Reproductive Health, National Institute for Medical Research, Mwanza, Tanzania

^8^Department of Anthropology, School of Arts and Sciences, Rutgers University, New Jersey, United States of America

* Corresponding author

**E-mail:** humphreymazigo@gmail.com

# Abstract

# Background

*Schistosoma haematobium* causes urogenital schistosomiasis and is widely distributed in Tanzania. In girls and women, the parasite causes Female Genital Schistosomiasis (FGS), a gynecological manifestation of schistosomiasis that is highly neglected and overlooked by public health professionals and policy makers. This study explored community members’ knowledge, attitudes and perceptions (KAP) on and health seeking behavior for FGS.

# Methods/Principal Findings

Using qualitative research methods—including 40 Focus Group Discussions (FGD) and 37 Key Informant Interviews (KII)—we collected data from 414 participants (F=50.7% [201], M=49.3% [204]) in nine (9) purposively selected districts of Zanzibar (4 districts) and northwestern Tanzania (5 districts). A purposive sample of participants was selected based on gender, age and position in the community. Most participants were aware of urogenitalurogenital schistosomiasis. Children were reported as the most affected group and blood in urine was noted as a common symptom especially in boys. Adults were also noted as a risk group due to their involvement in activities like paddy farming that expose them to infection. Most participants lacked knowledge of FGS and acknowledged having no knowledge that urogenital schistosomiasis can affect the female reproductive system. A number of misconceptions on the symptoms of FGS and how it is transmitted were noted. Adolescent girls and women presenting with FGS related symptoms were reported to be stigmatized, perceived as having a sexually transmitted infection (STI), and sometimes labeled as “prostitutes”. Health seeking behavior for FGS included a combination of traditional medicine, self-treatment and modern medicine.

# Conclusion/Significance

Community members living in two very different areas of Tanzania exhibited major, similar gaps in knowledge about FGS. Our data illustrate a critical need for the national control program to integrate public health education about FGS during the implementation of school- and community-based mass drug administration (MDA) and with improvement of water, sanitation and hygiene (WASH) facilities.

# Author summary

Female Genital Schistosomiasis (FGS) is a disease of the female reproductive system caused by infection with *Schistosoma haematobium*, a parasite acquired through skin contact with infested water. Although this disease is highly endemic in many parts of sub-Saharan Africa and associated with serious effects on reproductive and mental health, FGS is often neglected by public health professionals and policy makers. The knowledge, attitudes, and practices (KAP) of FGS in endemic communities—which is important for adherence to intervention measures—is unclear. This study used qualitative research methods to explore community members’ KAP on and health seeking behavior for FGS. Overall, community members knew about urogenitalurogenital schistosomiasis, but lacked knowledge of FGS. Misconceptions on its causes, symptoms and mode of transmission were common. Our study highlights the need for public health education to address FGS as part of community-based mass drug administration (MDA). In addition, MDA should be extended to women and adolescent girls in the communities in order to reduce the burden of FGS. For long-term impact of these interventions, improving the supply of water, sanitation and hygiene (WASH) is an essential part of the intervention package to end urogenital schistosomiasis and FGS.

# Introduction

The sub-Saharan Africa region carries the highest burden of schistosomiasis, accounting for 95% of the >230 million cases estimated to occur worldwide [1–3]. Approximately two-thirds of these cases are caused by *Schistosoma haematobium*, a diecious blood parasite acquired by skin contact with contaminated fresh water [1,2]. The parasite is endemic in many communities characterized by low socio-economic status with poor or inadequate water, sanitation and hygiene (WASH) infrastructure [4]. In endemic areas, all community members, irrespective of their age and gender, are at risk of infection whenever they contact with infested water sources [5–8]. However, specific gendered roles and norms may increase the risk of infection for some groups. For instance, women and girls of reproductive age (WGRA) perform household and other related activities—such as washing clothes and dishes, fetching water for domestic use, and paddy farming—that put them at high risk as they involve requisite contact with water [7,8]. In contrast, boys and men mainly contract the infection through swimming, farming, and fishing [8]. In both sexes, *S. haematobium* affects the urogenital and genital tracts [9]. Specifically, in an estimated 40-56 million women and girls, *S. haematobium* causes a gynecological disease referred to as Female Genital Schistosomiasis (FGS) [10,11].

Clinical manifestations of FGS include vaginal bleeding, vaginal itching, pain during sexual intercourse, and formation of sandy patches on the cervix and uterus [11–13]. FGS has been linked to increased susceptibility of HIV in women [14–17]. Untreated FGS can lead to infertility, miscarriage, ectopic pregnancies, and spontaneous abortions [14]. In addition, untreated FGS can lead depression and social stigma in girls and women who are perceived to have a sexually transmitted infection (STI) or who are struggling with infertility [18]. Yet, FGS remains highly neglected. For instance, as of the time of submission, FGS has not been included as part of schistosomiasis by the Global Burden of Diseases Study [19] and is largely ignored by public health professionals, policy makers and endemic communities [19–21]. In highly endemic areas, standard diagnostic equipment, treatment, skilled healthcare workers and prevention services are inadequate [14]. Moreover, most communities lack awareness about FGS and the diagnosis is not considered among adolescent girls and women having reproductive health issues [22].

Little is known about communities’ knowledge, attitudes, and practices (KAP) on and health seeking behavior for FGS. At the time of submission, only one study in Africa has assessed the community KAP on FGS [22]. It is generally accepted that proper KAP of endemic communities play a significant role in attaining sustainable disease control [23]. Moreover, design and implementation of effective behavioral change interventions to reduce risk and promote care-seeking require a clear understanding of the community’s knowledge and perception of the targeted disease. In that context, this study aimed to understand communities’ KAP on and health seeking behavior for FGS. We conducted the study in two parts of Tanzania with very disparate cultural and religious beliefs, but similar levels of *S. haematobium* endemicity (prevalence). In both areas, we also sought community recommendations on how best to promote community awareness about FGS and health seeking behavior among WGRA in Tanzania.

# Methods

**Ethical considerations**

This study received ethical approvals from the Lake Zone Institutional Review Board (certificate number MR/53/100/649), Zanzibar Health Research Ethics Committee (certificate number ZAHREC/03/PR/DEC/2020/29), and Weill Cornell Medicine (certificate number 20-07022381). Regional and district authorities where the study was conducted granted permission to enable the study to be conducted in their jurisdiction. The study team adhered to all ethical principles. Adult participants aged 18 years and above gave written informed consent. Participants below the age of 18 years gave their assent and their parents or guardians gave a written informed consent for them to participate in the study. Prior to data collection, the study team conducted sensitization meetings with responsible authorities in every study village/shehia to create awareness of the study and its data collection procedures. The study team-maintained anonymity of the study participants and confidentiality of the information provided. Individual identifiers were not used throughout the study. All participants were identified using code numbers.

## Study setting

This study was conducted between September 2020 and February 2021 in schistosomiasis-endemic districts in north-western Tanzania and Zanzibar (Fig 1). In north-western Tanzania, *S. haematobium* is endemic in communities located away from Lake Victoria shoreline (i.e. farther south and south-east of the lake) while *S. mansoni*—a parasite that causes intestinal schistosomiasis—is endemic in communities located along the lake’s shoreline (Fig 2) [24,25]. We conducted this study in 24 purposively selected villages located in districts lying within the *S. haematobium* transmission zone: Itilima, Maswa, Misungwi, Shinyanga Rural, and Kwimba (Fig 1) [26]. Geographically, these districts are characterized by permanent and seasonal fresh water rivers, marshes, swamps, and ponds which create a good living and breeding environment for *Bulinus* snail species—specifically *Bulinus nasutus*—which transmit *S. haematobium* [24]. Despite repeated rounds of mass drug administration (MDA) in these districts, some villages still experience high transmission of *S. haematobium* [24,25]. FGS is a public health concern in these districts and one study reported an average prevalence of 5% (ranging from 0-11%) [16].

In Zanzibar, the study was conducted in 16 purposively selected shehias (pl., smallest administrative areas in Zanzibar equivalent to wards) located in 7 schistosomiasis-endemic districts (Fig 2) [27]. The districts include North A, North B, Central, and West on Unguja Island as well as Wete, Chake Chake, and Mkoani on Pemba Island. At the time of submission, no study had been conducted to measure the burden of FGS in Zanzibar.

In sum, we purposively selected study sites located in *S. haematobium* transmission zones, particularly those that depend on and are located close to the open, permanent sources of water including man-made dams, marshes, swamps, and rivers. In selecting these sites, we also depended on the historical data from the district-level neglected tropical disease (NTD) control coordinators, which indicated that these communities had the highest burden of schistosomiasis. Finally, the selected sites had implemented school-based MDA programs using praziquantel (PZQ) as the main schistosomiasis control measure.

**Figure 1.** Study districts in A. in northwestern Tanzania and B. Pemba and Unguja, Zanzibar

**Figure 2.** Prevalence of schistosomiasis in Tanzania mainland and Zanzibar in 2017. The northwestern districts along Lake Victoria, the northern part of Unguja Island and the southern part of Pemba Island are highly endemic (Adapted from <https://espen.afro.who.int/tools-resources/search-maps>)

**Study design and recruitment of participants**

The study used qualitative research methods to allow participants to share their perspectives in light of their lived experiences and social context [28,29]. Using Focus Group Discussions (FGDs) [30–32] and Key Informant Interviews (KIIs) [33], we collected data from participants including adolescent boys and girls, adult men and women, older men and women, government leaders, and opinion leaders. The study team worked with the village chairpersons, village executive officers (VEOs), and shehas (pl., leaders of shehias in Zanzibar) to purposively select FGD participants based on age and gender (Table 1). Those who met the inclusion criteria were invited to participate in the study. FGDs were conducted with adolescent boys (15-20 years), adolescent girls (15-20 years), adult women (21-45 years), adult men (21-45 years), older men (≥46 years), older women (≥46 years), and opinion leaders (of different ages). Except for FGDs conducted with opinion leaders, which involved both male and female leaders, other FGDs were divided by gender. We conducted a total of 46 FGDs, 30 in north-western Tanzania and 16 in Zanzibar (Table 2). Key informants were purposively selected and invited to participate in the study based on the position they hold in the community. We conducted a total of 37 KIIs, 29 in northwestern Tanzania and 8 in Zanzibar (Table 3). Key informants included village chairpersons, VEOs, shehas, and opinion leaders (including sheikhs, pastors, traditional healers, and community health workers [CHWs]). In total, the study recruited 414 participants (Female=50.7% [201], Male=49.3% [204]) to take part in the FGDs and KIIs (Table 3).

**Table 1. Demographic characteristics of the study participants**

| **Variable** | **Female** | **Male** | **Total** |
| --- | --- | --- | --- |
| Age groups (in years) | | | |
| 15 - 20 | 63 | 60 | 123 |
| 21 - 45 | 104 | 93 | 197 |
| ≥46 | 43 | 51 | 94 |
| **Education level** | | | |
| Did not complete primary education | 22 | 6 | 28 |
| Primary | 79 | 90 | 169 |
| Secondary | 98 | 93 | 191 |
| College | 11 | 15 | 26 |
| **Marital status** | | | |
| Single | 82 | 70 | 152 |
| Widow | 28 | 0 | 28 |
| Married | 100 | 134 | 234 |
| **Total** | **210** | **204** | **414** |

**Table 2. Study participants, FGDs conducted, and study sites**

| **FGD code** | **Participants** | **Participants’ gender** | | **Village/Shehia** | **District** | **Location in Tanzania** |
| --- | --- | --- | --- | --- | --- | --- |
|  |  | **Male** | **Female** |  |  |  |
| 01 | Adolescent girls | 0 | 11 | Lagangabilili | Itilima | Northwestern Tanzania |
| 02 | Adolescent boys | 8 | 0 | Mwabulugu |  |  |
| 03 | Adolescent girls | 0 | 8 | Simiyu |  |  |
| 04 | Adolescent boys | 8 | 0 | Mhunze |  |  |
| 05 | Adult men | 8 | 0 | Ng’wang’wita |  |  |
| 06 | Adult women | 0 | 8 | Mwanunui |  |  |
| 07 | Adult women | 0 | 8 | Bumera |  |  |
| 08 | Adult men | 8 | 0 | Mwazimbi |  |  |
| 09 | Elder women | 0 | 8 | Mhunze |  |  |
| 10 | Elder men | 8 | 0 | Simiyu |  |  |
| 11 | Opinion leaders | 6 | 2 | Mwamtani |  |  |
| 12 | Opinion leaders | 6 | 2 | Itubilo |  |  |
| 13 | Adolescent boys | 8 | 0 | Masawe | Misungwi |  |
| 14 | Adolescent girls | 0 | 8 | Masawe |  |  |
| 15 | Adult women | 0 | 8 | Mwajombo |  |  |
| 16 | Adult men | 7 | 0 | Mwajombo |  |  |
| 17 | Adolescent girls | 0 | 8 | Manawa | Kwimba |  |
| 18 | Adolescent boys | 7 | 0 | Manawa |  |  |
| 19 | Adult women | 0 | 9 | Ibindo |  |  |
| 20 | Adult men | 6 | 0 | Ibindo |  |  |
| 21 | Adolescent girls | 0 | 7 | Ikwingwamanoti | Shinyanga Rural |  |
| 22 | Adolescent boys | 6 | 0 | Ikwingwamanoti |  |  |
| 23 | Adult women | 0 | 7 | Ikwingwamanoti |  |  |
| 24 | Adult men | 8 | 0 | Ikwingwamanoti |  |  |
| 25 | Opinion leaders | 8 | 1 | Ikwingwamanoti |  |  |
| 26 | Adolescent girls | 0 | 6 | Mwashegeshi | Maswa |  |
| 27 | Adolescent boys | 7 | 0 | Mwashegeshi |  |  |
| 28 | Adult women | 0 | 7 | Mwaneghele |  |  |
| 29 | Adult men | 6 | 0 | Mwaneghele |  |  |
| 30 | Opinion leaders | 6 | 3 | Mwaneghele |  |  |
| 31 | Adolescent girls | 0 | 8 | Mwera | West | Unguja Island, Zanzibar |
| 32 | Adult men | 11 | 0 | Kinuni |  |  |
| 33 | Adult women | 0 | 10 | Chaani Masingini | North A |  |
| 34 | Adult men | 12 | 0 | Kandwi |  |  |
| 35 | Adolescent boys | 9 | 0 | Kinyasini |  |  |
| 36 | Adult women | 0 | 8 | Bandamaji |  |  |
| 37 | Adult women | 0 | 8 | Kitope | North B |  |
| 38 | Adult women | 0 | 12 | Miwani | Central |  |
| 39 | Adolescent boys | 8 | 0 | Chambani | Mkoani | Pemba Island, Zanzibar |
| 40 | Adult women | 0 | 9 | Wambaa |  |  |
| 41 | Adult men | 8 | 0 | Mtambile |  |  |
| 42 | Adolescent girls | 0 | 9 | Uwandani | Chake Chake |  |
| 43 | Adult women | 0 | 8 | Mavungwa |  |  |
| 44 | Adult women | 0 | 8 | Kwale |  |  |
| 45 | Adult men | 9 | 0 | Wawi |  |  |
| 46 | Adult women | 0 | 8 | Kangagani | Wete |  |
|  |  | **178** | **199** |  |  | **Total** |

**Table 3**. Key informants, number of KIIs, and study sites.

| **KII code** | **Participants** | **Participants’ gender** | | **Village/Shehia** | **District** | **Location in Tanzania** |
| --- | --- | --- | --- | --- | --- | --- |
|  |  | **Male** | **Female** |  |  |  |
| 01 | Sheikh | M |  | Mhunze | Itilima | Northwestern Tanzania |
| 02 | CHW |  | F | Mhunze |  |  |
| 03 | CHW |  | F | Simiyu |  |  |
| 04 | Traditional healer | M |  | Mwanunui |  |  |
| 05 | VEO | M |  | Ng’homango |  |  |
| 06 | Village chairperson | M |  | Ng’wang’wita |  |  |
| 07 | Famous man | M |  | Sagata |  |  |
| 08 | Retail drug seller |  | F | Sagata |  |  |
| 09 | Famous woman |  | F | Sagata |  |  |
| 10 | Pastor | M |  | Nanga |  |  |
| 11 | Famous man | M |  | Tagawi |  |  |
| 12 | Famous woman |  | F | Mwamsheni |  |  |
| 13 | Traditional healer | M |  | Masawe | Misungwi |  |
| 14 | Village chairperson | M |  | Masawe |  |  |
| 15 | Pastor | M |  | Mwajombo |  |  |
| 16 | CHW |  | F | Mwajombo |  |  |
| 17 | VEO | M |  | Manawa | Kwimba |  |
| 18 | Retail drug seller |  | F | Manawa |  |  |
| 19 | Famous woman |  | F | Ibindo |  |  |
| 20 | Sheikh | M |  | Ibindo |  |  |
| 21 | Traditional healer | M |  | Ikwingwamanoti | Shinyanga Rural |  |
| 22 | VEO | M |  | Ikwingwamanoti |  |  |
| 23 | Famous man | M |  | Ikwingwamanoti |  |  |
| 24 | Retail drug seller | M |  | Ikwingwamanoti |  |  |
| 25 | CHW |  | F | Ikwingwamanoti |  |  |
| 26 | Pastor | M |  | Mwashegeshi | Maswa |  |
| 27 | Village chairperson | M |  | Mwashegeshi |  |  |
| 28 | Retail drug seller |  | F | Mwaneghele |  |  |
| 29 | Sheikh | M |  | Mwashegeshi |  |  |
| 30 | Sheha |  | F | Mwera | West | Unguja Island, Zanzibar |
| 31 | Sheha | M |  | Chaani Masingini | North A |  |
| 32 | Sheha | M |  | Kinyasini |  |  |
| 33 | Sheha | M |  | Kandwi |  |  |
| 34 | Sheha | M |  | Mtambile | Mkoani | Pemba Island, Zanzibar |
| 35 | Sheha | M |  | Wambaa |  |  |
| 36 | Sheha | M |  | Chambani |  |  |
| 37 | Sheha | M |  | Uwandani | Chake Chake |  |
|  |  | **26** | **11** |  |  | **Total** |

## Data collection

Data collection was conducted between September 2020 and February 2021. Before starting fieldwork, field supervisors and research assistants with qualitative research experience were recruited and retrained on how to collect qualitative data using FGDs and KIIs. All field supervisors and research assistants read, became conversant with, and participated in the pre-testing of the semi-structured topic guides that would later be used to collect data. In selecting venues for discussions and interviews, the research team chose places that did not have interruptions and which ensured privacy. All FGDs and KIIs were conducted at a time and venue suggested by the research team and approved by the participants. Except for FGDs with opinion leaders that were conducted by a team of both male and female research assistants, other FGDs and KIIs were led by research assistants of the same gender as participants. Each FGD was led by three research assistants, each playing a distinct role as either a moderator, a digital audio recorder operator, or a note taker. All FGDs and KIIs were conducted in Kiswahili—a native language of both the research assistants and participants—and recorded using a digital audio recorder with the participants’ written consent. A note taker made handwritten notes of key issues that came up in the FGDs. At the end of each day of data collection, the research team held a debriefing session to review the data, address issues that had emerged during the process, and plan for the next day.

**Data processing and analysis**

After data collection, all audio-recorded FGDs and KIIs were transcribed verbatim by a team of transcribers trained on research ethics and handling of qualitative data. AS and VJL verified the transcripts ensuring that they represented their audio versions. We coded the data transcripts using both deductive (concept-driven) and inductive (data-driven) coding approaches [34]. Transcripts were coded in Kiswahili to preserve the participants’ original concepts and meanings [35] which are likely to be lost during the translation process. Before coding the transcripts, DJM prepared a codebook with the initial set of deductive codes, drawing from the FGD and KII semi-structured guides. AS, VJL, and AK inductively coded six (6) transcripts (2 transcripts each, 1 FGD and 1 KII) to identify new codes and themes. Thereafter, DJM, AS, VJL, and AK discussed and refined the deductive and inductive codes and themes to produce a refined codebook. To ensure intercoder reliability [36], all transcripts were imported on the NVivo 12 Plus software and coded by AS and VJL using the refined codebook. Lastly, themes were grouped into categories and selected quotes were chosen—and translated to English—to illustrate major findings.

# Results

We present the results on urogenital schistosomiasis and FGS. For urogenital schistosomiasis, our analysis came up with six interlinked themes: awareness, perceived prevalence, aetiology, symptoms, modes of transmission, and groups of people at high risk of urogenital schistosomiasis infection. For FGS, our analysis came up with eight interlinked themes: awareness, aetiology and transmission, symptoms, women at higher risk of infection, associating FGS with other diseases or medical conditions, community perception of FGS infected women and girls, treatment seeking behavior for FGS, and interventions to mitigate the transmission of FGS.

## Awareness of urogenital schistosomiasis

Before delving into FGS, we explored community members’ awareness of schistosomiasis. The majority of the participants had heard about urogenital schistosomiasis and blood in urine was reported as a common symptom, especially among boys. Urogenital schistosomiasis was considered to be very prevalent, and in Zanzibar, it was ranked among the top three prevalent diseases. Although children were considered to be at higher risk of contracting the disease, women and men were equally mentioned to be at risk because of their involvement in water contact activities such as paddy farming. Transmission of schistosomiasis was often reported to be associated with water contact. Misconceptions that urogenital schistosomiasis was sexually transmitted were noted in both sites. Participants did not clearly understand the difference between aetiology (causes) and the modes of transmission of FGS. Major community perspectives on urogenital schistosomiasis in both parts of Tanzania are summarized in Table 4.

**Table 4. Community members’ knowledge and perceptions about urogenital** schistosomiasis in Northwestern Tanzania and Zanzibar

| **Results** | **Theme** | **Illustrative Quotations** |
| --- | --- | --- |
| Most participants were aware of urogenital schistosomiasis. | Participants’ sources of information about schistosomiasis included school, home, and the community | - “*I have heard about it at school, at home, and generally from our community.*” (FGD 02, Adolescent boys, Itilima). |
|  | Some participants were aware of schistosomiasis because they had been infected by it at some point in their lives | - “*I once tested* *[…] and was diagnosed with schistosomiasis*.” (KII 01, Sheikh, Itilima). - “*Yeah, I contracted schistosomiasis when I was still young, at the age of six, studying in primary school. I was infected and treated at home by my parents using herbal medicine. [Even then] Schistosomiasis was well known [by many people. In Kisukuma language, we call it* kisambale. *It is a disease that has existed for many years*.” (FGD 25, Opinion leaders, Shinyanga Rural). |
| Participants knew most of the symptoms of urogenital schistosomiasis | A few participants were aware of some of the common symptoms of urogenital schistosomiasis including muscle aches | - “*When someone is infected with schistosomiasis, they don’t feel well. They experience muscle aches. They also experience difficult in urination. At the end of urination, blood comes out.*” (FGD 34, Adult men, North A). |
|  | Most participants were aware of most of the chronic symptoms of urogenital schistosomiasis including abdominal/pelvic pain, difficulty passing urine, pain during urination, frequent urination, and blood in urine (haematuria). | - *“When you get infected with schistosomiasis, you start experiencing pain. At the end of urination, blood comes out. When you see a heavy bloody discharge blood, it is a sign that the infection has become chronic.”* (KII 35, Sheha, Mkoani). - *“Another symptom of schistosomiasis is abdominal pain.” (FGD 25, Opinion leaders, Shinyanga Rural).* - *“When you urinate, your urine is hot, it comes in small amounts, and it hurts [pain during urination]. Those are some of the symptoms of schistosomiasis.”* (FGD 06, Adult women, Itilima). - *“The stomach hurts a lot [abdominal pain]. And he/she [the infected person] urinates frequently. When peeing, there comes blood at the end.”* (FGD 05, Adult men, Itilima). - *“Some of the symptoms that a person infected with schistosomiasis […] shows are feeling severe pain during urination, drops of blood at the end of urination, and abdominal pain.”* (FGD 18, Adolescent boys, Kwimba). - “*If you get infected with schistosomiasis, you will definitely know that this is schistosomiasis. Your urine changes and blood appear in your urine at the end of urination. So, you will just know it is schistosomiasis.*” (FGD 34, Adult men, North A). |
| Schistosomiasis is perceived to be highly prevalent in endemic communities, though some observed improvements. | Most participants perceived that the infection rate of schistosomiasis was very high | - “*In my shehia, schistosomiasis is a serious problem.*” (KII 35, Sheha, Mkoani). - “*The information we receive from our experts […] tells us that schistosomiasis is still a problem. Topographically, our places have many valleys and basins where water accumulates. Our shehia has many freshwater sources. According to experts, fresh water sources are the main source of schistosomiasis. Given this nature of the environment [surrounded by freshwater rivers], schistosomiasis is an ever-present disease in our community.*” (KII 34, Sheha, Mkoani). - “*Schistosomiasis infection rate is so high because people use and trample on contaminated water […]. When [people] go to the health facility [for diagnosis], they find that they have contracted* kisambale *[schistosomiasis]*.” (FGD 09, Elder women, Itilima). |
|  | A few participants observed that urogenital schistosomiasis infections have declined mainly because of school-based MDA programs | - “*These days, fewer people urinate blood because children are treated in school*.” (KII 11, Famous man, Itilima). |
| All groups of people are at risk of being infected with urogenital schistosomiasis although their levels of risk differ | Young people, both male and female, are at higher risk compared to other groups | - “*Schistosomiasis infection rate is very high among us, young people. I can say, at around ninety percent*.” (FGD 02, Adolescent boys, Itilima). - “*Children are at risk [of being infected with schistosomiasis] because they have frequent contact with water. They are not careful. Our environment is wet but they sit anywhere. They bathe in rivers, not once, not twice, but many times in one day. Therefore, children are at higher risk [than other groups].*” (KII 34, Sheha, Mkoani). - “*I can say that schistosomiasis is a problem because it is a common disease, especially among children. Children are affected more than adults because they like playing in the ponds. […] and pond water is more likely to have schistosomiasis parasites*.” (FGD 27, Adult men, Maswa). - “*Schistosomiasis affects young people the most. [Young people] aged between fifteen and twenty years are very much affected. I think that their blood is prone to this disease. I am saying this because schistosomiasis troubled us [older men and women] too when we were young*.” (KII 22, VEO, Shinyanga Rural). |
|  | Although boys and girls are at high risk, girls have a relatively lower risk of infection than boys | - “*Girls are at a relatively lower risk than boys because they are not free to move around in the community. Boys have a higher risk than girls because they contact with contaminated water very frequently.*” (KII 34, Sheha, Mkoani). |
|  | Women are at risk because of doing domestic chores which involve contact with contaminated water | - “*Women are at risk because of the lack of clean and safe tap water […]. Women are responsible for fetching water for household use and they carry out most of the domestic chores. We are also responsible for preparing water for our husbands [e.g. water for taking a shower]. It is expected that we make sure, they get water. But where do we get it? Other groups at risk are paddy farmers and vegetable growers because they fetch water for irrigating their farms from the rivers.*” (KII 30, Sheha, West). |
| Most participants understood that the transmission of schistosomiasis occurs when people suffering from schistosomiasis contaminate freshwater sources with their excreta containing parasite eggs (which then hatch in water). | Urinating in freshwater sources (when infected with schistosomiasis) transmits schistosomiasis | - “*If a person infected with schistosomiasis urinates in the river and I contact that contaminated water, I will be infected with schistosomiasis.*” (FGD 39, Adolescent boys, Mkoani). - “*One of the people’s behavior here is that they urinate in rivers. Then, some of us go to the same rivers to take bath and wash clothes and utensils. In addition, having multiple sexual partnerships can also lead to being infected with schistosomiasis.*” (FGD 32, Adult men, West). - “*Contaminated water in the ponds may contribute [to the transmission of schistosomiasis]. An infected person may bath and then urinate in that water. If another person comes to bath in the same water, then they can be infected with schistosomiasis*.” (FGD 02, Adolescent boys, Itilima). |
| Most participants mentioned contact with contaminated fresh water as a key mode of transmission of schistosomiasis | People may be infected with schistosomiasis if they come into contact with contaminated fresh water | - “*Based on my understanding, schistosomiasis is a disease that is caused by parasites. Other people say [it is caused by] bacteria, but we can just say they are parasites. They are found in fresh water sources and particularly stagnant water. If you come into contact with this water, you will be infected with schistosomiasis.*” (KII 33, Sheha, North A). - “*Schistosomiasis is a disease that is caused by [coming into contact with] contaminated water from the rivers. It is a disease that causes pain during urination and blood appears in the urine at the end.*” (FGD 33, Adult women, North A). |
|  | Contact with contaminated freshwater may occur while farming in wet areas such as paddy fields | - “*You can be infected, for instance, through farming in the paddy fields where you come into contact with the already infested water flowing in from distant places and possibly passes through unsanitary places*…” (FGD 06, Adult women, Itilima). - “*Farming in the majaruba (paddy fields) is a risk factor. If the paddy field has those parasites [causing schistosomiasis] and the person is working in there, he can be infected.*” (KII 07, Famous man, Itilima). - “*People who like swimming in pond water can get infected with schistosomiasis parasites. Also, in this community, people are farmers and they get infected with schistosomiasis when they participate in agricultural activities in the wetland. When a person stands in the wetland, the parasite that causes schistosomiasis enters through their skin and continues to other parts of the body*.” (KII 17, VEO, Kwimba). |
|  | Contact with contaminated freshwater may occur when a person baths in pond water | - “*Schistosomiasis is caused by parasites that live in pond water. When you bathe in this water, you get infected with these parasites*.” (FGD 25, Opinion leaders, Shinyanga Rural). |
|  | Contact with contaminated freshwater may occur because of swimming or bathing in rivers or ponds | - “*Children are children. It is not possible for them to stop swimming in the rivers whenever they try to. Swimming in the rivers is their custom.*” (KII 34, Sheha, Mkoani). - “*Oftentimes*, *children like bathing in the ponds. And they play with mud. They play in dirty places because they are not aware that playing in such places puts them at risk of being infected with diseases [such as schistosomiasis].*” (FGD 02, Adolescent boys, Itilima). |
| A few participants held misconceptions that schistosomiasis is sexually transmitted or caused by salt consumption | Some people misconceived that schistosomiasis is transmitted through sexual intercourse | - “*At times, you can transmit schistosomiasis to another person through sexual intercourse [...]. If I am infected with schistosomiasis and then I have sex with another person, [I can transmit it to them].*” (FGD 10, Adult men, Itilima). - “*I have the same ideas as my fellow participant […]. Like he said, schistosomiasis is transmitted through sexual intercourse*.” (FGD 16, Adult men, Misungwi). |
|  | Some people misconceive that schistosomiasis is transmitted by sharing undergarments with an infected person | - “*Besides having sex with an infected person, people also speculate that sharing clothes, particularly undergarments, with an infected person can transmit schistosomiasis*.” (FGD 12, Opinion leaders, Itilima). |
|  | Some people misconceived that consuming a lot of salt causes schistosomiasis | - “*My father was telling me about my grandfather who died [long ago] that he [the grandfather] contracted schistosomiasis because he was eating salt*.” (FGD 26, Adolescent girls, Maswa). - “*[You get infected with] schistosomiasis when you eat too much salt. You know there are people who eat salt like sugar […]. When you eat salt frequently, you just eat salt every day […], you will be infected with schistosomiasis.*” (FGD 03, Adolescent girls, Itilima). |
| Some participants confused the causes of schistosomiasis and its aetiology | Mode of transmission (in this case contact with contaminated fresh water) confused with aetiology (fresh water parasitic worms) | - “*The main source of schistosomiasis is water. That is the source which has germs that cause schistosomiasis.*” (FGD 43, Adult women, Chake Chake). - “*[Schistosomiasis] is caused by playing with contaminated water frequently and bathing in the river or stream water flowing through the valley. That is, it, [it is caused] by taking a birth in dirty water. We should tell our children to stop playing in dirty water because that is the cause of schistosomiasis.*” (FGD 36, Adult women, North A). |

## Awareness of Female Genital Schistosomiasis

Most participants in both parts of Tanzania had never heard of FGS despite knowing about urogenital schistosomiasis, its urogenital symptoms, and risk factors for acquisition. Despite the same parasite typically causing both urinary and genital symptoms, perspectives were summarized well by a female respondent’s admission that “*I have never heard of it [schistosomiasis] in the female genitalia. I have heard of it in the bladder*” (FGD 07, Adult women, Itilima). A key informant in Zanzibar acknowledged that “*I have never heard about FGS* (KII 33, Sheha, North A). Despite having never heard about FGS, another respondent suggested that “*if that disease [urogenital schistosomiasis] stays in the body for a long time, it will evolve into another disease […] and can spread into a woman’s uterus*” (FGD 10, Adult men, Itilima).

A small number of participants mentioned hearing about schistosomiasis causing infertility but this was perceived as a rare event, with one woman qualifying her statement about this by saying that “*I heard this from people, not experts. I cannot lie, I have never heard this [FGS] from experts*” ( FGD 07, Adult women, Itilima).

## Aetiology and modes of transmission of Female Genital Schistosomiasis

Most of the study participants reported that they neither knew the aetiology of FGS nor its modes of transmission. As one of the female participants plainly acknowledged, “*I don’t know at all. I don’t know what causes FGS*” (FGD 07, Adult women, Itilima). There was confusion between aetiology and the mode of transmission. Participants related transmission of FGS with water contacts.

“*A woman can also be infected with schistosomiasis by bathing in the river or when washing clothes in the river while standing in water. This is the main reason for getting those germs. And, women are more infected than men because the anatomy of women’s reproductive system is different from that of men. This is a reason why women are more likely to be infected than men* (FGD 32, Adult men, West).

Despite relating FGS transmission with water contacts, they were not sure how urogenital schistosomiasis—which they were aware of—related to FGS. Other participants related the proximity of the urogenital bladder and the female reproductive tract as a source of infection to the female genitalia.

“*If the urinary bladder is affected, how can the vagina be spared while both are in the same area? How? The germs [that cause FGS] spread. They don’t enter and just stay in the urinary bladder or any other area where the urine passes. They spread [to other parts or organs]. The reproductive tract is close to the urinary bladder. They are neighbours. So, if you urinate blood and the organ you use is located in the same area, then germs will spread to the reproductive tract*.” (FGD 38, Adult women, Central).

“*You know the anatomy of a woman, right? When the parasites enter the woman’s [external genital organs], it is like they have entered the supermarket. They quickly spread to other parts. They can go to the reproductive tract and even the urogenital tract*.” (KII 23, Famous man, Shinyanga Rural).

Others guessed what they thought could be the causes of FGS and how it could be transmitted from one person to another. Most participants thought that FGS can be transmitted through sexual intercourse either from an infected male to a female or vice versa.

“*FGS can be caused by sexual intercourse. [This is] because during sexual intercourse, if you [a man] have parasites [that cause FGS] you can leave them in a woman. Those parasites will then infect her*.” (FGD 10, Adult men, Itilima).

“*I think as we said earlier, [FGS] can be spread even through sexual intercourse. So, I also think that even a woman can get this infection in her reproductive tract by engaging in unprotected sex with someone [male] who is infected with schistosomiasis parasites*.” (FGD 13, Adolescent boys, Misungwi).

“*When a woman is infected with schistosomiasis in her genital tract, she can infect her sexual partner or her husband. During sexual intercourse, she can infect him because those bacteria stay in the genital tract.*” (FGD 04, Adolescent boys, Itilima).

“*We can say that most adults get infected with schistosomiasis through sexual intercourse. That is the main source. If a woman is infected and the man is not, when they have sex, then the man will be infected*.” (KII 21, traditional healer, Shinyanga Rural).

Others reported that FGS could be transmitted by coming into contact with vapor emitted from urine or other body fluids from a person infected with the disease.

“*A woman can be infected through urine in the toilet while urinating. As she urinates, if someone else with a similar problem [infected with FGS] urinated there, then there is vapor that normally comes out. That vapor enters the female genitals directly. This is one way that may contribute to FGS infection*.” (FGD 02, Adolescent boys, Itilima).

“*An infected woman can infect another person when, for instance, she sits on a place and then another person comes to sit on the same place later. The infected woman can leave some tiny droplets on that place. When uninfected person sits on the same place, the bacteria [that cause FGS] are transmitted through the droplets into his/her body*.” (FGD 05, Adult men, Itilima).

## Symptoms of Female Genital Schistosomiasis

When asked about the symptoms of FGS, most of the participants admitted that they did not know about them. Most of those who attempted to respond to this question linked the symptoms of urogenital schistosomiasis with symptoms of FGS.

“*I think someone experiences severe pain especially lower back pain. Sometimes they feel headache*.” (FGD 06, Adult women, Itilima).

“*Feeling pain in private parts, blood in urine, and pain during urination. I think these are the symptoms [of FGS]*.” (FGD 32, Adult men, West).

“*What I know is that when a woman [who is infected with FGS] urinates, blood will appear in urine at the end urination*.” (FGD 08, Adult men, Itilima).

Some men appeared confident when explaining the symptoms of FGS. One man described “*frequent severe abdominal pain and bright red period blood*” (FGD 08, Adult men, Itilima) as the main symptoms of FGS. Another man explained the progression of FGS symptoms by saying, “*First a woman feels pain. Second, a woman fails to perform sexual intercourse. Third, the woman’s genital organs hurt*.” (FGD 05, Adult men, Itilima).

Symptoms that most respondents mentioned frequently included abdominal pain, pain during sexual intercourse, pain during urination, blood in urine (haematuria), miscarriage and irregular menstruation. Other symptoms that were mentioned albeit a few times included itching during urination, vaginal pain, fatigue, pain during sexual intercourse, frequent urination (polyuria), loss of libido (sex drive), headache, burning sensation, infertility, sterility, severe pain before menstruation, uterine damage, leg pain, vaginal discharge (sometimes containing pus), pus in urine (pyuria), and vaginal sores.

Although most of the symptoms mentioned were indeed of FGS, it became clear that most participants guessed or derived their responses on their knowledge of (the symptoms of) urogenital schistosomiasis, intestinal schistosomiasis and sexually transmitted infections (STIs) such as gonorrhea and syphilis. This became clear particularly when respondents were probed to explain more about symptoms of FGS that they had mentioned. For example, when asked to explain what causes irregular menstruation, participants said it could be because of stomach-ache, urogenital schistosomiasis, frequent sexual intercourse, traumatic events, menopause, and change of environment.

## Women at higher risk of Female Genital Schistosomiasis

Most participants perceived every female person in their communities as being at risk of being infected with FGS. As one female participant noted, “*This disease can infect every woman regardless of age or age group. It can infect even the elderly*” (FGD 07, Adult women, Itilima). However, WGRA were considered as being at higher risk than other age groups of women, followed by girls and older women. Their risk of FGS infection was associated with engaging in water contact activities and sexual intercourse.

*Women and girls between fifteen and thirty-five years [are most at risk of schistosomiasis infection]. Girls play with contaminated water in the rivers. Others [women and girls] wash clothes in contaminated water in the rivers*.” (FGD 33, Adult women, North A)*.*

“*Girls of reproductive age—not like me [she was in her 40s]—go to the river, bath in contaminated water and end-up being infected with schistosomiasis*.” (FGD 36, Adult women, North A).

To explain this difference in levels of risk, most respondents interestingly mentioned sexual intercourse and involvement water contact activities (such as rice farming in paddy fields) as the two main factors that put WGRA at higher risk of FGS infection than other groups of women.

“*They are usually infected through sexual intercourse. If a woman is an adult, she will certainly engage in sexual activity.*” (FGD, 10, Adult men, Itilima).

“*Those who are affected more are girls who are sexually active. Those who have multiple sexual partners. This contributes to their risk of being infected with FGS.*” (FGD 35, Adolescent boys, North A).

“*For a woman of reproductive age, FGS is transmitted through sexual intercourse with her husband or having sex with another partner. This is the* *only way she can be infected.*” (KII 09, Famous woman, Itilima).

“*Women who come into contact with stagnant water, for instance, when working in the majaruba (paddy fields) can be infected with schistosomiasis*.” (KII 06, Village chairperson, Itilima).

## Associating Female Genital Schistosomiasis with other diseases or medical conditions

Many respondents admitted that they did not know how FGS was associated with other diseases or medical conditions such as HIV/AIDS and other STIs, cervical cancer, ectopic pregnancy, miscarriage, infertility and sterility. Several others made educated guesses using their knowledge of urogenital schistosomiasis or perceptions of FGS. For instance, two participants related FGS with HIV/AIDS by reasoning that:

“*When someone is infected with schistosomiasis the entire urogenital system gets bruises. When she engages in sexual intercourse, she can be infected with other diseases such as HIV/AIDS*.” (FGD 05, Adult men, Itilima).

“*HIV/AIDS and schistosomiasis are different disease diseases according to experts. But they infect the same tract. When you get schistosomiasis, you also get HIV/AIDS. You will now have two diseases and the problem becomes severe*.” (KII 34, Sheha, Mkoani).

In the same topic, another participant explained the relationship between FGS and noted that a person with FGS, “*can easily get sexually transmitted infections, for example, I don’t know!! Yes, is called gonorrhoea. Yes, one can get all these diseases which affects the sexual organs*” (FGD 39, Adolescent boys, Mkoani).

Relating FGS and cervical cancer, a man explained that “*schistosomiasis causes wounds which can become malignant. When they become malignant, it can be a problem. [Cervical] cancer can be in the form of those wounds. This means the disease has become chronic and caused cancer*” (FGD 05, Adult men, Itilima). Other participants, however, did not know how these diseases relate. As one participant revealed, “*One can be affected by cervical cancer. But I don’t have much knowledge of how this can happen; I don’t have much time and understanding about it, I am just guessing*” (FGD 39, Adolescent boys, Mkoani). Others reasoned that these two diseases did not relate. One participant reasoned that “*schistosomiasis affects the female genital tract especially the urogenital bladder and the cervix. Cancer is present in the cervix, a place where schistosomiasis doesn’t reach*” (FGD 04, Adolescent boys, Itilima).

Other participants related FGS with infertility. One participant explained that when FGS affects “*the entire sexual reproductive system. That is when you get the problem of infertility*” (FGD 05, Adult men, Itilima). Others related reasoned that FGS could cause miscarriage. One informant explained how this can happen: “*because those ‘insects’ [parasites causing FGS] attach the pregnancy. When they attach the pregnancy, they lead to miscarriage*” (KII 07, Famous man, Itilima).

## Community perception of Female Genital Schistosomiasis infected women and girls

Participants in both parts of Tanzania, mainland and Zanzibar, reported that FGS infected women and girls are often labeled as “*prostitutes*” or unfaithful in their intimate relationships. They also reported that if the person infected with FGS is a young girl, community members would think that she has started having sex at a young age and view her as promiscuous. Some of the participants explained these individual and community perceptions.

“*What I know is that in our community, people will think that she [a woman or girl with symptoms of FGS] is a prostitute.*” (KII 01, Sheikh, Itilima).

“*[On observing the symptoms of FGS], the husband will not think it is [Female Genital] Schistosomiasis. He will think that she is promiscuous and that is why she got that infection. He will think that she had sex with someone infected with an STI. And they will break up.*” (FGD 33, Adult women, North A).

*In our village, if one [a male sexual partner] hears that their girlfriend is bleeding [showing symptoms of FGS], they will be afraid. They will suspect that she has a sexually transmitted infection and think that they will also be infected. So, they will break up [with the girl] and chase her away.* (FGD 14, Adolescent girls, Misungwi).

However, other participants reported that as schistosomiasis, particularly urogenital schistosomiasis, was becoming better known, stigmatization was decreasing.

“*Stigmatization may occur. However, these are isolated cases because schistosomiasis is now regarded as normal problem.*” (KII 07, Famous man, Itilima).

Participants reported that if the infected person is a married woman, just like for girls, community members might perceive them as being promiscuous and unfaithful to their partners. Interestingly, they might also think that they have contracted STIs.

“*They won’t think that this woman has schistosomiasis. They will think that she has a sexually transmitted infection.*” (FGD 06, Adult women, Itilima).

If the FGS infected woman happens to be an older woman—and since older women are regarded as less sexually active—this infection will be regarded as other normal diseases. In other words, older women who are infected with FGS are not stigmatized.

“*If an older woman has those symptoms [of FGS], the community cannot claim that she has been infected by another person. They will simply say she got infected [because of other reasons other than sexual activity].*” (FGD 06, Adult women, Itilima).

## Treatment seeking behavior for Female Genital Schistosomiasis

In north-western Tanzania, participants reported that community members often rely on traditional healers or self-treatment with natural herbs to manage their illness before going to a biomedical facility to seek diagnosis and treatment. In Zanzibar, community MDA was reported as the main source of schistosomiasis treatment. They also mentioned traditional healers and health facilities as the source of treatment.

“*In our setting, many people rush to the traditional healers. Others go to the dispensaries, health centres, and hospitals. But the decision really depends a person’s level of education [level of awareness about treatment options and efficacy].*” (FGD 05, Adult men, Itilima).

“*Some people get medication over the counter. They buy it or get it for free from another person and treat themselves [without being diagnosed].*” (KII 07, Famous man, Itilima).

“*The government is aware of this disease [schistosomiasis]. Therefore, it provides drugs in different areas. There are distributors in our community. People who feel that they are infected can go and get the drugs.*” (FGD 35, Adolescent boys, North A).

“*I don’t know where the drugs [for schistosomiasis] are sold. I cannot afford to buy drugs. But we are used to getting them from our Sheha*.” (FGD 39, Adolescent boys, Mkoani).

## Interventions to mitigate the transmission of FGS

We asked study participants to suggest the best interventions that could be implemented at different levels to mitigate the transmission of FGS. We categorized their suggestions into two groups: (1) interventions aimed at improving the availability of FGS services for women suffering from this infection, and (2) interventions aimed at integrating FGS, Sexual and Reproductive Health (SRH), STIs and cervical cancer screening services.

### Improving the availability of Female Genital Schistosomiasis services

Participants suggested that in order to mitigate the suffering from undiagnosed and untreated FGS among women and girls, the government—either alone or with support from Non-governmental Organizations (NGOs)—needs to improve the availability and delivery of FGS services. They suggested three forms of interventions. First, they suggested increasing diagnostic capacity for FGS at local dispensaries and health centres, making this care accessible to patients who currently must travel long distances to seek these services at the hospitals.

“*In our place, diagnostic services are not available. Health facilities that have laboratories are far from here. If you want to do a checkup today, you have to travel far. And because the economy is not good, even if you don’t feel well, you will keep postponing to go. But if the services are located near to us, if you feel [you are infected with FGS], you will go for the services.*” (KII 01, Sheikh, Itilima).

Second, they suggested that authorities should recruit more health workers. They also suggested that the government should train more health workers so that they can have skills to provide services in a friendly manner.

“*The government should employ more health workers. For instance, we have few doctors and nurses here. If we have enough health workers, people will get good health care and services within a short time*.” (FGD 26, Adolescent girls, Maswa)

“*The government should plan to train health workers with skills to provide counseling [and other services well] professionally. Not these rude [service providers]. Sometimes you came across a mean service provider. Maybe they are in a bad mood that day. You approach them for a service that they know is available [at their health facility], but they refer you to Nyambiti hospital [located far from this village] where the service is more expensive*.” (FGD 18, Adolescent boys, Kwimba).

Third, they suggested the need to ensure medications are available for patients to access and use when they go to the health facilities.

“*The other thing is that you find that the medications are out of stock. I don’t understand how the medications get out of stock. Maybe they provide services to many people or sell them? I can’t prove this. But we usually face the shortage of essential medications.*” (KII 10, Pastor, Itilima).

“*The government should ensure medications are available. The stock should be increased so that doctors can prescribe medications to their patients here [at the health centre] instead of referring them to Maswa [District Hospital]. For example, if a pregnant woman comes here, they should be able provide her with all services, no referring her to Maswa [District Hospital]*.” (FGD 26, Adolescent girls, Maswa).

### Integrating female genital schistosomiasis services, sexual reproductive health, sexually transmitted infections and cervical cancer screening services

Participants suggested that creating awareness of FGS, cervical cancer, and HIV was the key intervention that would promote women’s SRH. Participants suggested a number of ways in which education would improve women’s awareness of the need for, and access to these services. First, they suggested that people need to be educated about FGS including its symptoms, and effects so that they can make informed decisions about health care seeking whenever they sense its symptoms.

“*To be motivated [to access FGS services], people should be educated about FGS, its symptoms, and effects. If you know these aspects, you will sense that may be with these symptoms, I have this or that problem [FGS] and that if I don’t act [go to the hospital], I will end up with certain effects.*” (FGD 11, Opinion leaders, Itilima).

“*The main intervention that can eliminate schistosomiasis is education. The community should be educated to adhere to interventions so that schistosomiasis can be eliminated. Here is Mwera, only drug distributors are educated. Other community members are not. It is good to educate all people so that they can know what schistosomiasis is.*” (FGD 31, Adolescent girls, West).

“*People should be sensitized that this disease is caused this way. And that if you see such symptoms you rush to the healthcare facility where you will be given certain specific types of treatment*.” (FGD 19, Adult women, Kwimba).

Connected to this, participants also suggested that authorities can organize meetings where women and girls can be educated by health professionals about the variety of threats to women’s SRH.

“*What should be done is to call a meeting and educate them [women and girls] about the problem [SRH problem] and its effects, and to encourage them to visit the health facility to find out what the problem is if they have certain symptoms.*” (FGD 02, Adolescent boys, Itilima).

Others suggested that health workers or community health workers (CHWs) can conduct house-to-house sensitization meetings to educate people about SRH issues.

“*When you gather people together [like in a meeting], they tend to conceal their problems. They don’t want everyone to know about their health problems. So, the best way is to visit every household.*” (FGD 08, Adult men, Itilima).

Another benefit of the education would be to help people understand the symptoms, mode of transmission, and effects of both FGS and STIs as a way to fight stigma leveled against women and girls with these infections. In the case of FGS, participants suggested that health workers should also be educated to treat FGS-infected patients like other patients. With no or less stigmatization, these women and girls can then easily access health services. As one leader explained,

“*Knowledge will help people to become self-aware and understand the symptoms [of FGS]. Once she understands the symptoms, she can be free to share with people who will be able to advise her on how and where to get treatment. So, there will be no stigma.*” (FGD 11, Opinion leaders, Itilima).

Women also emphasized the importance of training health workers so that they treat FGS-infected patients like other patients instead of stigmatizing them.

“*They should treat them well like any other patients who come with other diseases. If you love someone and show her that you love her, she can feel happy and come [for services at the facility] without any worries.*” (FGD 06, Adult women, Itilima).

Others suggested that a mobile clinic would be beneficial in fighting stigma against FGS.

“*Because many women are scared of going to the health facilities for testing because someone will see them. If the services are brought directly to the village […] many people will get tested*.” (FGD 06, Adult women, Itilima).

# Discussion

Our data demonstrated a strong need for health education, particularly about FGS. In both parts of Tanzania, despite a broad and relatively accurate community knowledge about urogenital schistosomiasis, almost nothing was known about FGS. Further, women suffering with symptoms of FGS are often stigmatized by other community members. Addressing gaps in knowledge are critical in order to achieve community participation and adherence to control measures against FGS. Moreover, community members provided practical suggestions for how education can be provided. The strong similarities from communities with very different cultures suggests that our findings are broadly generalizable throughout Tanzania and beyond.

FGS is highly overlooked and neglected by health professionals, local and global policy makers [10,21,37]. Consistently, in Tanzania, there have been very few community-based studies which have addressed FGS in adolescent girls and women [16,38–40] and none have assessed the KAP of these groups in relation to FGS. Our findings from the FGDs and KIIs indicate that the majority of the participants knew about urogenital schistosomiasis but had never heard of FGS and were not aware that schistosomiasis may affect a woman’s reproductive system. Similarly, in a previous study in Ghana, none of the community members who participated in FGDs reported having heard of FGS [22] and only 18.9% of participants agreed that urogenital schistosomiasis could cause reproductive health disease [41]. In Egypt, it was similarly found that endemic communities had knowledge about urogenital schistosomiasis as a disease but did not believe that it had reproductive health effects [42].

Consistent with findings from these other studies, the majority of our study participants neither knew the cause of FGS nor its modes of transmission. Misconceptions on the modes of transmission were noted with sexual intercourse, vapor coming from pit latrines, and urinating on the same spot with an infected person being the most common responses. Our findings regarding the confusion surrounding modes of transmission are confirmed by a similar study in Ghana [22]. In addition, participants struggled to list symptoms related to FGS. Though participants guessed or derived their responses from their knowledge of the urogenital schistosomiasis and STIs, some of the mentioned symptoms were directly related to FGS by chance. In Ghana, though adult and adolescent girls discussed genital symptoms they experienced, they did not relate their symptoms to schistosomiasis [22].

Women in the study communities were considered to be at risk of FGS but WGRA age were considered at higher risk compared to older women. WGRA were reported to engage in activities, which brought them into regular contact with contaminated water including economic activities such as paddy farming in the wetlands and domestic chores such as washing clothes in contaminated rivers and ponds. These gendered roles which require women to regularly come into contact with cercariae-contaminated water increase women’s risk of being infected with schistosomiasis [7,43]. In rural schistosomiasis-endemic communities in Africa, lack of alternative sources of water forces them to use contaminated water sources for domestic and agricultural purposes [8,22]. To achieve sustainable control of schistosomiasis, specifically FGS, integrating MDA with WASH should be part of the development agenda. Improvement in water supply in endemic communities has been reported to decrease the risk of being infected with urogenital schistosomiasis [44–46]. Behavioral changes that limit exposure is one of the recommended preventive measures against schistosomiasis. However, without improving the availability of clean and safe water in endemic communities, it is difficult for people to change their water contact behaviors.

Socially, FGS symptoms in women and adolescent girls can led to stigma and may affect health-seeking behavior. Participants in both areas of Tanzania reported that women and girls presenting the symptoms of FGS or STIs are labeled as “prostitutes” within their communities. Similarly, girls with genital symptoms of FGS were accused of promiscuity and in health facilities they were likely to be referred for STI treatment [22]. Our findings and those from Ghana [22] clearly indicate that there is a need to implement public health interventions at the community and health facility levels to, first, create awareness about FGS, its symptoms/clinical signs, diagnosis, management, and prevention and, second, encourage women and girls to seek for treatment at health facilities.

Community members’ health seeking behavior was reported to rely on traditional healers, self-treatment using local herbs and biomedical treatment. Before seeking modern medicine, community members preferred to seek treatment from either traditional healers or self-treatment using either herbs or buy medicine from drug shops. Long queues at the health facilities were cited as a reason for opting for traditional medicine and self-treatment. Similar findings were reported in northwestern Tanzania [47,48] and Ghana [49]. Perceptions that urogenital schistosomiasis does not have serious negative health impacts, is a disease that can heal spontaneously without treatment and is a normal part of life all led to community members opting for traditional medicine [47,48]. In Ghana, adolescent girls cited stigmatization as a barrier to seeking care from health facilities and opted for home or self-treatment [22]. Previous findings showed that women who experienced symptoms such as bleeding after sexual intercourse were reluctant to seek treatment because of the fear of being associated with having an STI [50]. Partly, this could be related to lack of FGS knowledge among healthcare providers. Actually, one of the major gaps in addressing FGS is inadequate capacity of healthcare providers to effectively identify, refer, diagnose and treat women and girls [21].

In the FGDs and KIIs participants suggested practical strategies that are likely to be effective in controlling FGS in their communities, in addition to MDA using PZQ and improvement of WASH [44,46,51]. The following were recommended: (1) improve availability of diagnostic services at the level of dispensaries and health centres, (2) increase the number of skilled healthcare providers who are able to offer services in a friendly manner, (3) improve availability and accessibility of medicine, (4) offer health education to motivate adolescent girls and women to seek care, and (5) provide in-service training to healthcare providers so they can offer FGS services without stigmatizing women and girls who present with genital symptoms. Furthermore, participants recommended integration of FGS services with other services offered in SRH such as STI preventions and cervical cancer screening. Similar recommendations were provided by international global health professionals [20].

This study’s findings revealed that the surveyed communities had adequate knowledge about urogenital schistosomiasis. For example, transmission was well known to be associated with water contacts and the symptom of blood in urine was commonly described. High risk activities including water contact among adults and children were mostly known. However, misconceptions were also common, with some people reporting schistosomiasis as a STI. Cumulatively, the gaps of knowledge related to schistosomiasis observed in the present study communities have also been observed by previous studies in urogenital schistosomiasis-endemic areas [22,47,48]. In Magu and Shinyanga districts, northwestern Tanzania [47,48] and Ghana [22,41], urogenital schistosomiasis was considered as a STI.

Previous studies in Zanzibar [52] and Northwestern Tanzania [48] noted that community members associated urogenital schistosomiasis with boys more than girls. These beliefs were also present in our study. Biomedically, urogenital schistosomiasis is highly prevalent among young populations, with children aged 8-15 years carrying the highest burden of the disease [9]. In relation to gender, conflicting evidence exist [43]; some studies have reported females to be at the highest risk and carry the highest burden while other studies have reported the opposite [8,43]. In parasitological surveys, blood in urine detected by either visual or urine reagent dipstick is more often reported in boys than in girls [53,54]. The relationship between schistosomiasis and boys has been repeatedly reported in endemic countries [22,48]. Other endemic communities consider the infection (blood in urine) as normal in boys and believe that it does not have a health impact [52]. These results define the difference in exposure to risk environment between females and males when carrying out different activities in contaminated water sources [8]. Confirming results from the FGDs and KIIs, parasitological surveys have previously reported that the selected districts are highly endemic for urogenital schistosomiasis [24] and transmission occurs seasonally [27,55]. High transmission occurs between March and May, the period of the long rainy season, and lower transmission occurs during the rest of the year [55]. In mainland Tanzania, control of schistosomiasis targets mainly school children and is offered at school [24]. Repeated rounds of treatment have resulted in a decline in prevalence and intensity of infection. However, a single intervention will not eliminate the disease.

In spite of the relevant findings from our study, we acknowledge a number of limitations. First, the findings are based on the participants’ narratives and experiences and may thus represent skewed perspectives by those willing to participate in the discussions. Second, we did not conduct parasitological and gynecological examinations to ascertain the burden of urogenital schistosomiasis and FGS. Instead, we relied on previous studies that had confirmed high prevalence of *S. haematobium* in the study communities. Third, some of the probes included in the data collection tools may have shaped the responses of the participants, especially on FGS. In spite of these limitations, our findings demonstrate inadequate knowledge about urogenital schistosomiasis and more specifically about FGS in different parts of Tanzania.

# Conclusions and recommendations

Our findings demonstrate that communities living in known *S. haematobium* endemic areas of Tanzania have a relatively accurate knowledge about urogenital schistosomiasis but knowledge about FGS is lacking. Misconceptions on the aetiology and modes of transmission of urogenital schistosomiasis and FGS were common. Community members recognized the need for being educated about these diseases. Our data emphasize the urgent need for public health interventions to focus on improving community awareness of FGS, which in turn will reduce stigma and improve women and girls’ health seeking behavior. Furthermore, integration of FGS services into existing SRH services with skilled healthcare providers will enhance the health seeking behavior of women and girls. Managing FGS will be an important aspect of the intervention. Not only will it lead to decreased gynecological symptoms and stigma faced by women and girls but will reduce the risk of contracting HIV and other STIs. Lastly, for successful control of schistosomiasis and FGS, inclusion of women and girls in annual MDA is highly recommended to reduce disease prevalence and related morbidities while we pursue the ultimate goals of improving the supply of WASH to achieve schistosomiasis elimination.

# Acknowledgements

The authors would like to thank the study participants for their time and responses without which this paper would not have been possible. We also thank the regional, district, shehia and village authorities where the study was conducted for granting the permission and providing every support to successfully conduct this study. Finally, we are grateful to Prof. Jennifer A. Downs of the Center for Global Health, Weill Cornell Medicine, New York, United States of America for comments and reviews on this paper.

# Author contributions

**Conceptualization:** Humphrey D. Mazigo and Jennifer A. Downs

**Data curation:** Anna Samson, Valencia J. Lambert, Agnes Kosia, Dunstan J. Matungwa.

**Formal analysis:** Humphrey D. Mazigo, Anna Samson, Valencia J. Lambert, Agnes Kosia, Dunstan J. Matungwa.

**Funding acquisition:** Humphrey D. Mazigo.

**Investigation:** Humphrey D. Mazigo, Anna Samson, Valencia J. Lambert, Agnes Kosia, Dunstan J. Matungwa.

**Methodology:** Humphrey D. Mazigo, Anna Samson, Valencia J. Lambert, Agnes Kosia, Dunstan J. Matungwa.

**Project administration:** Humphrey D. Mazigo, Anna Samson.

**Supervision:** Humphrey D. Mazigo, Anna Samson.

**Writing – original draft:** Humphrey D. Mazigo.

**Writing – review & editing:** Humphrey D. Mazigo, Anna Samson, Valencia J. Lambert, Agnes Kosia, Dunstan J. Matungwa, Rachel Murphy, Deogratias D. Ngoma.

**Data availability**

Qualitative data collected in this study contains information which can make participants identifiable and therefore data cannot be shared publicly. However, data are available on special request

**Competing interest**

The authors declare that there are no competing interests

**Funding**

This work was supported by the Task Force for Global Health, Coalition for Operational Research on Neglected Tropical Diseases (NTD-SC-208.2D). HDM received additional funding from UK Foreign Commonwealth and Development Office (FCDO) through the Accelerating the Sustainable Control and Elimination of Neglected Tropical Diseases (Ascend) programme (FCDO grant number PO-8374). The views expressed in this publication are those of the authors and not necessarily those of the funding agencies. Its contents are solely the responsibility of the authors and do not necessarily represent the official views of the supporting offices.

# References

1.	World Health Organization. Schistosomiasis. Geneva: WHO; 2020. Available: https://www.who.int/news-room/fact-sheets/detail/schistosomiasis [Accessed 24 March 2021]

2.	World Health Organization. Ending the neglect to ttain the sustainable development goals: A Road map for neglected tropical diseases 2021-2030. Geneva: WHO; 2020. Available: https://apps.who.int/iris/bitstream/handle/10665/332094/WHO-UCN-NTD-2020.01-eng.pdf?ua=1 [Accessed 23 March 2021)

3.	Rollinson D, Knopp S, Levitz S, Stothard JR, Tchuem Tchuenté L-A, Garba A, et al. Time to set the agenda for schistosomiasis elimination. Acta Trop. 2013;128: 423–440. doi:10.1016/j.actatropica.2012.04.013

4.	Hotez PJ, Fenwick A, Savioli L, Molyneux DH. Rescuing the bottom billion through control of neglected tropical diseases. Lancet. 2009;373: 1570–1575. doi:10.1016/S0140-6736(09)60233-6

5.	Hotez PJ, Kamath A. Neglected tropical diseases in sub-Saharan Africa: Review of their prevalence, distribution, and disease burden. Cappello M, editor. PLoS Negl Trop Dis. 2009;3: e412. doi:10.1371/journal.pntd.0000412

6.	Mugono M, Konje E, Kuhn S, Mpogoro FJ, Morona D, Mazigo HD. Intestinal schistosomiasis and geohelminths of Ukara Island, Northwestern Tanzania: Prevalence, intensity of infection and associated risk factors among school children. Parasit Vectors. 2014;7: 612. doi:10.1186/s13071-014-0612-5

7.	Hotez PJ. Empowering women and improving female reproductive health through control of neglected tropical diseases. PLoS Negl Trop Dis. 2009;3: e559. doi:10.1371/journal.pntd.0000559

8.	Sow S, de Vlas SJ, Stelma F, Vereecken K, Gryseels B, Polman K. The contribution of water contact behavior to the high Schistosoma mansoni infection rates observed in the Senegal River Basin. BMC Infect Dis. 2011;11: 198. doi:10.1186/1471-2334-11-198

9.	Ross AGP, Bartley PB, Sleigh AC, Olds GR, Li Y, Williams GM, et al. Schistosomiasis. N Engl J Med. 2002;346: 1212–1220. doi:10.1056/NEJMra012396

10.	Hotez PJ, Engels D, Gyapong M, Ducker C, Malecela MN. Female genital schistosomiasis. N Engl J Med. 2019;381: 2493–2495. doi:10.1056/NEJMp1914709

11.	Kjetland EF, Kurewa EN, Ndhlovu PD, Midzi N, Gwanzura L, Mason PR, et al. Female genital schistosomiasis - a differential diagnosis to sexually transmitted disease: genital itch and vaginal discharge as indicators of genital Schistosoma haematobium morbidity in a cross-sectional study in endemic rural Zimbabwe. Trop Med Int Heal. 2008;13: 1509–1517. doi:10.1111/j.1365-3156.2008.02161.x

12.	Poggensee G, Feldmeier H, Krantz I. Schistosomiasis of the female genital tract: Public health aspects. Parasitol Today. 1999;15: 378–381. doi:10.1016/S0169-4758(99)01497-0

13.	Kjetland EF, Ndhlovu PD, Mduluza T, Gomo E, Gwanzura L, Mason PR, et al. Simple clinical manifestations of genital Schistosoma haematobium infection in rural Zimbabwean women. Am J Trop Med Hyg. 2005;72: 311–9. doi:10.4269/ajtmh.2005.72.311

14.	Norseth HM, Ndhlovu PD, Kleppa E, Randrianasolo BS, Jourdan PM, Roald B, et al. The colposcopic atlas of schistosomiasis in the lower female genital tract based on studies in Malawi, Zimbabwe, Madagascar and South Africa. Downs JA, editor. PLoS Negl Trop Dis. 2014;8: e3229. doi:10.1371/journal.pntd.0003229

15.	Kjetland EF, Ndhlovu PD, Gomo E, Mduluza T, Midzi N, Gwanzura L, et al. Association between genital schistosomiasis and HIV in rural Zimbabwean women. AIDS. 2006;20: 593–600. doi:10.1097/01.aids.0000210614.45212.0a

16.	Downs JA, van Dam GJ, Changalucha JM, Corstjens PLAM, Peck RN, de Dood CJ, et al. Association of Schistosomiasis and HIV Infection in Tanzania. Am J Trop Med Hyg. 2012;87: 868–873. doi:10.4269/ajtmh.2012.12-0395

17.	Sturt AS, Webb EL, Francis SC, Hayes RJ, Bustinduy AL. Beyond the barrier: Female genital schistosomiasis as a potential risk factor for HIV-1 acquisition. Acta Trop. 2020;209: 105524. doi:10.1016/j.actatropica.2020.105524

18.	Litt E, Baker MC, Molyneux D. Neglected tropical diseases and mental health: A perspective on comorbidity. Trends Parasitol. 2012;28: 195–201. doi:10.1016/j.pt.2012.03.001

19.	Vos T, Barber RM, Bell B, Bertozzi-Villa A, Biryukov S, Bolliger I, et al. Global, regional, and national incidence, prevalence, and years lived with disability for 301 acute and chronic diseases and injuries in 188 countries, 1990–2013: A systematic analysis for The Global Burden of Disease Study 2013. Lancet. 2015;386: 743–800. doi:10.1016/S0140-6736(15)60692-4

20.	Engels D, Hotez PJ, Ducker C, Gyapong M, Bustinduy AL, Secor WE, et al. Integration of prevention and control measures for female genital schistosomiasis, HIV and cervical cancer. Bull World Health Organ. 2020;98: 615–624. doi:10.2471/BLT.20.252270

21.	Christinet V, Lazdins-Helds JK, Stothard JR, Reinhard-Rupp J. Female genital schistosomiasis (FGS): from case reports to a call for concerted action against this neglected gynaecological disease. Int J Parasitol. 2016;46: 395–404. doi:10.1016/j.ijpara.2016.02.006

22.	Kukula VA, MacPherson EE, Tsey IH, Stothard JR, Theobald S, Gyapong M. A major hurdle in the elimination of urogenital schistosomiasis revealed: Identifying key gaps in knowledge and understanding of female genital schistosomiasis within communities and local health workers. PLoS Negl Trop Dis. 2019;13: e0007207. doi:10.1371/journal.pntd.0007207

23.	Mwanga JR, Lwambo NJS. Pre- and post-intervention perceptions and water contact behavior related to schistosomiasis in northwestern Tanzania. Acta Trop. 2013;128: 391–398. doi:10.1016/j.actatropica.2012.09.017

24.	Mazigo HD, Nuwaha F, Kinung’hi SM, Morona D, de Moira AP, Wilson S, et al. Epidemiology and control of human schistosomiasis in Tanzania. Parasit Vectors. 2012;5: 274. doi:10.1186/1756-3305-5-274

25.	Clements ACA, Brooker S, Nyandindi U, Fenwick A, Blair L. Bayesian spatial analysis of a national urinary schistosomiasis questionnaire to assist geographic targeting of schistosomiasis control in Tanzania, East Africa. Int J Parasitol. 2008;38: 401–415. doi:10.1016/j.ijpara.2007.08.001

26.	Tanzania Ministry of Health and Social Welfare. Report on urinary schistosomiasis: National questionnaire baseline survey in Tanzania Mainland. Dar es Salaam: Schistosomiasis Control Initiative (SCI) and Ministry of Health and Social Welfare, Tanzania Mainland; 2010.

27.	Lwambo NJS, Savioli L, Kisumku UM, Alawi KS, Bundy DAP. Control of Schistosoma haematobium morbidity on Pemba Island: Validity and efficiency of indirect screening tests. Bull World Health Organ. 1997;75: 247–52.

28.	Cleland JA. The qualitative orientation in medical education research. Korean J Med Educ. 2017;29: 61–71. doi:10.3946/kjme.2017.53

29.	Mason J. Qualitative researching. 2nd ed. London: SAGE Publishing; 2002.

30.	Kitzinger J. Qualitative research: Introducing focus groups. BMJ. 1995;311: 299–302. doi:10.1136/bmj.311.7000.299

31.	Kitzinger J. The methodology of focus groups: The importance of interaction between research participants. Sociol Heal Illn. 1994;16: 103–121. doi:10.1111/1467-9566.ep11347023

32.	Hennink MM. Focus group discussions. Oxford: Oxford University Press; 2014.

33.	Gilchrist VJ, Williams RL. Key informant interviews. In: Crabtree BF, Miller WL, editors. Doing Qualitative Research. Thousand Oaks, CA: SAGE Publishing; 1999. pp. 71–88.

34.	Azungah T. Qualitative research: deductive and inductive approaches to data analysis. Qual Res J. 2018;18: 383–400. doi:10.1108/QRJ-D-18-00035

35.	Dey I. Qualitative data analysis: A user-friendly guide for social scientists. London and New York: Routledge. London: Routledge; 2005.

36.	O’Connor C, Joffe H. Intercoder reliability in qualitative research: Debates and practical guidelines. Int J Qual Methods. 2020;19: 160940691989922. doi:10.1177/1609406919899220

37.	Kjetland EF, Leutscher PDC, Ndhlovu PD. A review of female genital schistosomiasis. Trends Parasitol. 2012;28: 58–65. doi:10.1016/j.pt.2011.10.008

38.	Poggensee G, Saria M, Kiwelu I, Richter J, Krantz I, Feldmeier H. Schistosomiasis of the lower reproductive tract without egg excretion in urine. Am J Trop Med Hyg. 1998;59: 782–783. doi:10.4269/ajtmh.1998.59.782

39.	Poggensee G, Kiwelu I, Weger V, Göppner D, Diedrich T, Krantz I, et al. Female genital schistosomiasis of the lower genital tract: Prevalence and disease‐associated morbidity in Northern Tanzania. J Infect Dis. 2000;181: 1210–1213. doi:10.1086/315345

40.	Swai B, Poggensee G, Mtweve S, Krantz I. Female genital schistosomiasis as an evidence of a neglected cause for reproductive ill-health: a retrospective histopathological study from Tanzania. BMC Infect Dis. 2006;6: 134. doi:10.1186/1471-2334-6-134

41.	Yirenya-Tawiah DR, Ackumey MM, Bosompem KM. Knowledge and awareness of genital involvement and reproductive health consequences of urogenital schistosomiasis in endemic communities in Ghana: a cross-sectional study. Reprod Health. 2016;13: 117. doi:10.1186/s12978-016-0238-5

42.	Talaat M, Watts S, Mekheimar S, Farook Ali H, Hamed H. The social context of reproductive health in an Egyptian hamlet: A pilot study to identify female genital schistosomiasis. Soc Sci Med. 2004;58: 515–524. doi:10.1016/j.socscimed.2003.09.018

43.	Hotez P, Whitham M. Helminth infections: A new global women’s health agenda. Obstet Gynecol. 2014;123: 155–160. doi:10.1097/AOG.0000000000000025

44.	Grimes JET, Croll D, Harrison WE, Utzinger J, Freeman MC, Templeton MR. The Relationship between Water, Sanitation and Schistosomiasis: A Systematic Review and Meta-analysis. McGarvey ST, editor. PLoS Negl Trop Dis. 2014;8: e3296. doi:10.1371/journal.pntd.0003296

45.	Tanser F, Azongo DK, Vandormael A, Bärnighausen T, Appleton C. Impact of the scale-up of piped water on urogenital schistosomiasis infection in rural South Africa. Elife. 2018;7. doi:10.7554/eLife.33065

46.	Grimes JE., Croll D, Harrison WE, Utzinger J, Freeman MC, Templeton MR. The roles of water, sanitation and hygiene in reducing schistosomiasis: a review. Parasit Vectors. 2015;8: 156. doi:10.1186/s13071-015-0766-9

47.	Angelo T, Kinung’hi SM, Buza J, Mwanga JR, Kariuki HC, Wilson S. Community knowledge, perceptions and water contact practices associated with transmission of urinary schistosomiasis in an endemic region: a qualitative cross-sectional study. BMC Public Health. 2019;19: 703. doi:10.1186/s12889-019-7041-5

48.	Mwanga JR, Magnussen P, Mugashe CL, Gabone RM, Aagaard-Hansen J. Schistosomiasis-related perceptions, attitudes and treatment-seeking practices in Magu district, Tanzania: Public health implications. J Biosoc Sci. 2004;36: 63–81. doi:10.1017/s0021932003006114

49.	Danso-Appiah A, De Vlas SJ, Bosompem KM, Habbema JDF. Determinants of health-seeking behavior for schistosomiasis-related symptoms in the context of integrating schistosomiasis control within the regular health services in Ghana. Trop Med Int Heal. 2004;9: 784–794. doi:10.1111/j.1365-3156.2004.01267.x

50.	Ahlberg BM, Mwangi R, Poggensee G, Feldmeier H, Krantz I. ‘Better infection than hunger’: A study of illness perceptions with special focus on Urogenital Schistosomiasis in Northern Tanzania. African Sociol Rev. 2003;7: 18–34.

51.	WHO Expert Committee on the Control of Schistosomiasis and World Health Organization. Prevention and control of schistosomiasis and soil-transmitted helminthiasis: Report of a WHO expert committee. WHO Technical Report Series. Geneva: World Health Organization; 2002. Available: https://apps.who.int/iris/bitstream/handle/10665/42588/WHO_TRS_912.pdf?sequence=1&isAllowed=y [Accessed 25 March 2021]

52.	Person B, Ali SM, A’Kadir FM, Ali JN, Mohammed UA, Mohammed KA, et al. Community knowledge, perceptions, and practices associated with urogenital schistosomiasis among school-aged children in Zanzibar, United Republic of Tanzania. Pullan RL, editor. PLoS Negl Trop Dis. 2016;10: e0004814. doi:10.1371/journal.pntd.0004814

53.	Angelo T, Buza J, Kinung’hi SM, Kariuki HC, Mwanga JR, Munisi DZ, et al. Geographical and behavioral risks associated with Schistosoma haematobium infection in an area of complex transmission. Parasit Vectors. 2018;11: 481. doi:10.1186/s13071-018-3064-5

54.	Poggensee G, Krantz I, Nordin P, Mtweve S, Ahlberg B, Mosha G, et al. A six-year follow-up of schoolchildren for urogenital and intestinal schistosomiasis and soil-transmitted helminthiasis in Northern Tanzania. Acta Trop. 2005;93: 131–140. doi:10.1016/j.actatropica.2004.10.003

55.	Webbe G. The transmission of Schistosoma haematobium in an area of Lake Province, Tanganyika. Bull World Health Organ. 1962;27: 59–85.

# Supporting information
