# Supplementary material for: “We know about schistosomiasis but we know nothing about FGS”: A qualitative assessment of knowledge gaps about female genital schistosomiasis among communities living in Schistosoma haematobium endemic districts of Zanzibar and Northwestern Tanzania
Source: PLoS Negl Trop Dis. 2021 Sep 30;15(9):e0009789. doi: 10.1371/journal.pntd.0009789 (PMC8509863; doi:10.1371/journal.pntd.0009789)
Supplement: Attachment — Submitted filename: PLOS NTD_Responses to reviewers comments.docx [file pntd.0009789.s002.docx]

**Responses to reviewers’ comments**

Dear Editor

We thank the reviewers for taking time to review this manuscript. Their critical review, comments, edits, and suggestions improved the quality of this manuscript. Below are our responses to reviewers’ comments starting with reviewer 1, then reviewer 2 and finally reviewer 3. We have worked on the comments that reviewer 3 put in in the manuscript using track changes. Based on the comments of all the reviewers, we have also edited others parts of the manuscript where we thought that their comments were relevant although they had not indicated those parts.

| **S/no** | | **Reviewers’ comments** | **Response to reviewers’ comments** |
| --- | --- | --- | --- |
| **Reviewer 1** | | | |
| 1 | | A well-conducted qualitative study using focus group discussion and key informants’ interviews and in which the objectives are covered adequately with a large group of informants. | We thank the reviewer for this observation |
| 2 | | line 32 - Should be written Females and Males, respectively + Write n=201 and n=204, respectively | Correction done. We have also added the number of participants from mainland Tanzania and Zanzibar as was suggested by another reviewer |
| 3 | | Lines 127-130 - Figure 2 placed before Figure 1. The figures (maps – made for another purpose) are confusing. Suggest to prepare new maps for the specific purpose of this manuscript. | We have deleted Figure 2 as it is outdated |
| 4 | | Line 135 - Relatively low – in the general population, or the general female or age specific population? With such a low prevalence, how well should the population, then be expected to be knowledgeable about schistosomiasis in general and FGS specifically | The prevalence of S. haematobium, based on detection of eggs in urine samples among adolescent girls and women, is between 8%-57% and varies between villages in north-western Tanzania. The high prevalence of S. haematobium give indication that FGS could be a public health problem. However, it is neglected and not considered among the diseases which can lead to genital symptoms. Healthcare workers knowledge about FGS is also very poor (our manuscript on this topic is under review). We therefore cannot say with certainty that its prevalence is low. There are number of issues relating to underreporting and underdiagnosis. |
| 5 | | Line 137- In the abstract it says 4 districts of Zanzibar | The study was conducted in 7 districts in Zanzibar (4 districts on Unguja Island and 3 districts on Pemba Island) and 5 districts in northwestern Tanzania. We have edited the abstract and the methodology part of the paper accordingly. |
| 6 | | Lines 146-147 - Hence, a selected, and maybe not fully representative study population, being exposed to MDA programs, and as a result received information about schistosomiasis. What about populations not being targeted in the programs, probably even lower level of health literacy. That could have been interesting to assess as well. | In mainland Tanzania, MDA focus only in school aged children and no community MDA against schistosomiasis. In Zanzibar, MDA includes both community members and school aged children within a school environment. A combination of the two study sites offered an opportunity also to include areas where MDA is done and areas where MDA do not include adult members (some of whom were the study participants) |
| 7 | | Line 150 - In the figures, stated as Figure 1a and 1b, respectively. | Correction done to indicate the figures |
| 8 | | Line 152 - Confusing – also Figure 2 contains two maps, 2a and 2b, respectively | We have deleted Figure 2 |
| 9 | | Line 159 - In the abstract, FGD, and not FGDs, - KII likewise | Correction done |
| 10 | | Line 164 - It will give more detailed information if data about education level and marital status are presented in accordance to the three age-groups (and gender), and by use of percentages | We agree. Correction done |
| 11 | | Line 164 - What were the inclusion criteria, apart from age? Any exclusion criteria? | We have added information on the inclusion and exclusion criteria in the sub-section entitled “study design and recruitment of participants” |
| 12 | | Lines 170-172 - Suggest to place table 2 and 3 as supplements – not that important data | We agree. We have submitted them as supplementary documents |
| 13 | | Line 184 - Data were collected between… | Correction done |
| 14 | | Line 216 - Suggest to start with presentation of demographics (Table 1). | We have presented the demographics at the beginning of the results section |
| 15 | | Lines 217-219 - Which terms were used in communication with the participants in the FGDs and KII. Were those themes already identified and applied in a guide for the FGD and KII in order to ensure a semi-structured approach? | Yes, these themes were reflected in the open-ended questions that we put in the FGD and KII guides |
| 16 | | Lines 219-222 – likewise | Yes, these themes were reflected in the open ended questions that we put in the FGD and KII guides |
| 17 | | Line 227 - What were the other two? HIV, STIs? | Correction done |
| 18 | | Lines 232-233 - !!! See comment above (lines 217-222) | Correction done |
| 19 | | Table 4 (line 235-236) - A bit confusing. Theme in Table 4 seems not necessarily to be the same as themes in text (p.12, 216-219). Also confusing what is means by Results versus Theme in table 4 | Thanks for this observation. We have changed the wording in the first and second columns of the table. Instead of “results” the first column indicates the “themes” as they were identified in the analysis. And instead of the “themes” the second column presents the summary of the participants’ views.  In addition to this renaming, we have moved some of the participants’ views and quotes to other parts of the table where they fit most. |
|  | | Line 240 - How was FGS presented to the participants in the first place? As female genital schistomiasis, FGS or in another way in the opening question. What was the opening question? That must quite essential for assessment of awareness. If presented in clinical terms, most local people will not be able to recognize the subject. Same goes for other clinical terms, in particular using abbreviations. | Our approach was that we started with general knowledge about urogenital schistosomiasis to understand the community knowledge about schistosomiasis, how is transmitted, how it is perceived, any control measures within the community.  Then, we introduced “Female Genital Schistosomiasis” – starting by asking if they have ever heard about it and if so what it means and what could be its effects including which organs are affected by it. Then slowly by we began probing for details. We presented Female Genital Schistosomiasis in a non-technical language and we didn’t use the abbreviation. |
| 20 | | Line 214 - 216 - How is that – when there is no knowledge about the existence of FGS? (same goes for the other FGS themes) | As we present in the manuscript, not all participants had no knowledge about FGS. Some heard little knowledge about it and as described in our results, participants linked their knowledge on urogenital schistosomiasis with FGS. From this point, we continued probing about different thematic areas related to FGS. Our results clearly show, little knowledge in different aspects of FGS. Based on this, we certain that we have obtained evidence which clearly shows that FGS knowledge is low in the settings where we conducted this study. |
| 21 | | Line 451 - Likewise, how can FGS treatment seeking behaviour be assessed when there is no basic knowledge about existence of FGS? | We asked this question to the participants who indicated that they had some knowledge of FGS (whether they had heard about it or drew their knowledge from their experience with urogenital schistosomiasis). |
| 22 | | line 718 - Jennifer A. Downs is stated as contributing author to study conceptualization, but not stated as co-author | We have deleted Jennifer’s name from the author contributions section. But we have acknowledged her support in the conceptualization of the project that has led to, among other things, this paper, as well as for her comments, reviews and suggestions on the early drafts of this paper. |
| 23 | | In general, many Abbreviations/acronyms in the manuscript are used- are they all necessary | Indeed, we have used many abbreviations in this manuscript. However, most of them are commonly used. And for clarity, we have provided their full form where we use them for the first time. But here are some of the steps we have taken to reduce the number of abbreviations.  1) Instead of using the abbreviation WGRA which is not commonly used, we have replaced it with the noun phrase “girls and women of reproductive age” which is commonly used, particularly by the WHO.  2) We have deleted the abbreviation “PZQ” (an abbreviation of praziquantel) which was appearing only one time in the manuscript. Instead of using this abbreviation, we use its full form “praziquantel”.  3) In the “data processing and analysis” section, we used abbreviations of authors when indicating their roles in data processing and analysis. We have replaced these abbreviations (AS, VJL, AK, and DJM) with words “second, third, fourth and last author” so that they can easily be recognised using the order of authorship instead of abbreviations.  4) We have deleted other abbreviations including VEO, CHW and NGO and replaced them with their full form wherever they appear in the manuscript. |
| 24 | | The study addresses a neglected, but important, manifestation of a neglected tropical disease. It is important to include qualitative research methods in the overall assessment of FGS. The study confirms that health education about FGS among health care professionals as well as communities in S. haematobium endemic area is warranted to raise awareness towards essential transmission, prevention and treatment aspects. Most communities are familiar with abbreviation terms such as STIs and HIV related to reproductive health, but not to FGS - not even in S. haematobium endemic areas- across geographical regions, as exemplified in this multi-site study. | We thank the reviewer for this observation. |
| **25** | Overall results and key findings are well presented. | | Thank you |
| **26** | The qualitative results support the conclusion that FGS is indeed a neglected disease manifestation of high public health relevance in major parts of rural Africa | | Thank you |
| **Reviewer 2** | | | |
| 1 | | Data is presented clearly and comprehensively in the paper | Thank you |
| 2 | | Overall, this is an informative and important paper which adds to the much-needed evidence base about FGS in the community. It is an impressive sample size for qualitative work and represents a huge amount of time and effort. My comments are minor and suggest areas where additional clarity may help. | We thank the reviewer for this observation |
| 3 | | Abstract and author summary are clear. | Thank you |
| 4 | | Line 76: WGRA - is this commonly used? I have seen WRA (women of reproductive age) used more frequently. | Indeed, “women of reproductive age” is the most commonly used phrase with an abbreviation “WRA”. We have deleted the noun phrase “women and girls of reproductive age” and replaced it with “girls and women of reproductive age” which is oft-used, particularly by the WHO. |
| 5 | | Line 87: FGS can lead to | Correction done |
| 6 | | Line 98 - 99: Not sure about the statement “proper KAP”… I would suggest a less judgmental word than “proper”. Also, there is evidence suggesting knowledge alone does not change behaviour - but is one of many factors. | Correction done. We have replaced the word “proper” with “good.” |
| 7 | | Methods In the ethical considerations, did you have any cases where adult literacy was a problem and if so, how did you indicate assent to participate? | For participants who could not read and write, a consent/assent form was read aloud to them before they provided an oral consent/assent and signed using a thumbprint. We have added details on this in the ethical statement section. |
| 8 | | In the study design, why such variation in sample between the # of FGD e.g., 30 in NW Tanzania and 16 in Zanzibar? And # of IDI - 29 in NW Tanzania and 8 in Zanzibar? IT is clear that you identified more villages in NW Tanzania, but it isn’t clear how you determined how many IDI / FGD per site. | In the “Data collection and sample size” sub-section of the methodology, we have added the description on how we reached the sample size after attaining the point of saturation. |
| 9 | | Another question - this is quite a large sample for a qualitative study and wondering at what point you reached saturation? Did you plan to do so many IDI and FGD from the beginning? Or were any of these added until saturation was reached? Some comment about saturation would be helpful if/how it played a role in your sample size. | In the “Data collection and sample size” sub-section of the methodology, we have added the description on how we reached the sample size after attaining the point of saturation. |
| 10 | | Tables 2 and 3 are very clear and helpful. | Thank you |
| 11 | | Data collection Data collection was clearly outlined, as was data processing and analysis | Thank you |
| 12 | | Table 4 - is it possible to **id** potential respondents here - particularly the opinion leaders? Also line 279? Line 299? Line 374? Line 378? The terms of ‘famous man’ and ‘famous woman’ are unclear | Thank you for this suggestion. We have replaced the word “famous” with “influential” in all parts of the manuscript where it was appearing. |
| 13 | | Line 347 - this heading seems strange- men can’t be at any risk for FGS - so women have a “higher risk” than whom? Girls? Maybe “high risk” is a better term here? | Editing done. We now use the phrase “high risk” instead of “higher risk” |
| 14 | | The results are comprehensive and there may be some places where less quotes could be highlighted, even though they are interesting, picking fewer illustrative quotes may be good in some circumstances and would reduce length. It would also help the reader to include whether the site is Pemba or NW Tanzania. Without stating it, the reader must refer to the table. This would also help to illustrate the similar themes that arose in both sites. | a) Since we have a large number of FGDs and KIIs, we tried to ensure that we get good quotes from most FGDs and KIIs. We did this as a way of demonstrating that we worked on all our data rather than having a few quotes from a few FGDs and KIIs and seems as though we cherry-picked a few quotes to suit our objectives. This is the reason we have “many quotes” but with a good purpose of showing the extensiveness of our data. b) For every quote, we have added the description to show whether it is from North-western Tanzania or Zanzibar (Unguja or Pemba Island). Thank you for this suggestion. |
|  | | Given the lack of availability of Praziquantel and/or its prohibitive cost for some community members when purchased in a pharmacy, it is surprising that you did not hear more about this in your dataset. Is PZQ readily available in Tanzania to be purchased in the pharmacy? Can the authors provide a bit more context on this? | Praziquantel is available in mainland Tanzania and used for school-based mass treatment and in Zanzibar, is available to school children and adult members through community drug distributors. However, many of the health facilities including public facilities do not stock praziquantel. Some of the Community drug shops/community pharmacies serving in rural areas do stock PZQ and community members buy from these shops |
|  |  | In these areas of Tanzania, is cervical cancer screening common? Did the study probe as to why respondents thought it would be good to combine FGS with HPV/ cervical cancer screening? Indeed, it has been suggested in the global literature, but assume that study respondents wouldn’t have access to that information | Occasionally, cervical cancer screening is done when conducting campaigns targeting women in different areas of the country, especially in mainland Tanzania. In health facilities, when suspected, palp smears are done.  We noted in Zanzibar that there is a dedicated group of healthcare workers responsible for cervical cancer screening in the communities. Partly, this can explain the response of participants on this question.  N.B. We are presenting this information in a different manuscript that we currently working on. |
|  | | Line 685 - perhaps adding a comment about ease of reinfection? | Correction done |
| **Reviewer 3** | | | |
| 1 | | Are the objectives of the study clearly articulated with a clear testable hypothesis stated?  The objectives of the study are clearly articulated as follows:-  == This study aimed to understand communities’ KAP on and health seeking behaviour for FGS.  == We also sought community recommendations on how best to promote community awareness about FGS and health seeking behaviour among WGRA in Tanzania. | Thank you |
| 2 | | -Is the study design appropriate to address the stated objectives? == Yes, this is a qualitative research study using FGDs and KIIs to address the objectives. | Thank you |
| 3 | | -Is the population clearly described and appropriate for the hypothesis being tested? == It would be good to know some more details about the selection criteria of the study population. What were inclusion and exclusion criteria? | We have added the inclusion and exclusion criteria in the sub-section “Study design and recruitment of participants.” |
| 4 | | -Is the sample size sufficient to ensure adequate power to address the hypothesis being tested? = While the sample size seems large for a qualitative study, it is not clear, how the sample size was determined. If by saturation, it would be good if the authors can describe how saturation was reached and assessed. | In the “Data collection and sample size” sub-section of the methodology, we have added the description on how we reached the sample size after attaining the point of saturation. |
| 5 | | -Were correct statistical analysis used to support conclusions? == No (or only some descriptive) statistics were used in this qualitative research paper, which is fine. | Thank you |
| 6 | | -Are there concerns about ethical or regulatory requirements being met? == What was the procedure for child assenting? Was written assent provided? | One day before data collection, children aged 15-17 years were identified as potential participants through the community leaders. They were informed about the study objectives and given informed assent form to read and ask questions. They were also asked to inform their parents/guardians about the study and requested to come with them on the day of data collection for consent form signing.  Children were recruited after written informed assent and consent were received |
| 7 | | Does the analysis presented match the analysis plan? == No analysis plan is presented. | We have a sub-section of the methodology entitled “Data processing and analysis” in which we explain how we approached the analysis of our data. |
| 8 | | -Are the results clearly and completely presented? == The results are nicely structured and presented in tables and quotes. | Thank you |
| 9 | | -Are the figures (Tables, Images) of sufficient quality for clarity? == Table 2 and Table 3 could be moved to supporting information. == Table 4 is huge, but is very well structured and contains important results. == Figure 1 and 1 b are fine. == Figure 2 and 2b are outdated (from 2017) and since the manuscript is not about schistosomiasis elimination, I suggest to delete these figures. | - We agree, we are now presenting table 2 and 3 as supporting information  - We have deleted Figure 2 |
| 10 | | Are the conclusions supported by the data presented?  Yes  -Are the limitations of analysis clearly described? Yes  -Do the authors discuss how these data can be helpful to advance our understanding of the topic under study? Yes  -Is public health relevance addressed? Yes | Thank you |
| 11 | | Please see my specific comments and track-changes in the attached Word document that the authors may consider to enhance clarity.) | Thank you for these comments. We have worked on them to improve the clarity of the manuscript. |
| 12 | | Mazigo et al present a very nice paper, which highlights very interesting and important aspects regarding community knowledge and perceptions about urogenital schistosomiasis and female genital schistosomiasis. The manuscript is very well structured and written using good quality English throughout. The qualitative research study is very well conducted, including a relatively large number of participants from different areas of the United Republic of Tanzania, and differing in sex, age and position in community. The findings are nicely presented in tables and manuscript text. | Thank you |
| 13 | | 1) Please indicate the study type in the title- | We have indicated this in the title |
|  | | How many participants were from Zanzibar and how many from TZ mainland? | We have indicated how many participants were from Zanzibar and how many were from mainland Tanzania |
| 14 | | FGS is the disease, not the infection. This is incorrectly presented several times throughout the manuscript. | Thank you. We have corrected this. |
| 15 | | Mostly, it should read urogenital schistosomiasis and not urinary schistosomiasis, please see my track-changes in the text. | Thank you. We have corrected this. |
| 16 | | The authors may consider and present that in Zanzibar, MDA is directed to schools and communities. Hence, women and adolescent girls are targeted. | Thank you. We have corrected this. |
| 17 | | In Zanzibar, there are few hotspots and many low-prevalence shehias within the districts; the overall prevalence is very low. Hence, I think it is not appropriate to generalize districts as highly endemic. The figures 2a and 2b in my view are outdated and do not reflect the current situation on the ground. For more up to date information, please see: Trippler L, Ame SM, Hattendorf J, Juma S, Abubakar S, Ali SM, et al. Impact of seven years of mass drug administration and recrudescence of Schistosoma haematobium infections after one year of treatment gap in Zanzibar: Repeated cross-sectional studies. PLoS Negl Trop Dis. 2021;15(2): e0009127. Epub 2021/02/13. | -We have deleted Figure 2.  -And we have cited Tripper et al. 2021. Thanks for this tip. |
| 18 | | It will be good to describe how the participant sample size was determined. If based on saturation, the process to reach saturation should be described. It will also be good, if the authors can describe on more detail which participants were eligible. | In the “Data collection and sample size” sub-section of the methodology, we have added the description on how we reached the sample size after attaining the point of saturation. |
|  | | Please check whether the data availability statement is in line with PLOS NTD requirements-Check | We have rewritten the data availability statement in accordance with PLOS NTD requirements. Thank you. |
